# Effectiveness of *Glycyrrhiza uralensis* extract on periodontal pathogens: a randomized controlled clinical trial

**DOI:** 10.1186/s12903-025-06172-2

**Published:** 2025-05-24

**Authors:** Yu-Rin Kim, Seoul-Hee Nam

**Affiliations:** 1 Department of Dental Hygiene, Silla University, 140 Baegyang-daero, 700beon-gil, Sasang-gu, 46958 Busan, South Korea; 2 Department of Dental Hygiene, College of Health Sciences, Kangwon National University, 346 Hwangjo- gil, Dogye-up, Samcheok, 25945 Republic of Korea

**Keywords:** Gingivitis, Mouthwash, *Glycyrrhiza uralensis*, Microbiota, Clinical study

## Abstract

**Background:**

This study aimed to evaluate the possibility of using a mouthwash containing *Glycyrrhiza uralensis* (*GU*) extract as an oral health improvement material to prevent periodontal disease using the clinical parameters and the changes in bacteria that cause periodontal disease.

**Methods:**

A randomized, double-blind, controlled study was conducted on 60 patients who visited M dental clinic located in Busan, South Korea. The subjects were patients who agreed to complete the questionnaire, who were not included in the exclusion criteria, and had 16 or more remaining teeth. The patients were divided into two groups: the saline gargle solution group, which consisted of 30 patients; and the *GU* extract group, which consisted of 30 patients. Scaling was performed to ensure the same oral conditions between the two groups. After 1 week, 15 mL each of the gargle solution (saline and *GU* extract) was applied once a day for 30 s for 5 days. For the periodontal clinical parameters, O’Leary index, plaque index (PI), gingival index (GI), and periodontal-disease-related bacteria in subgingival plaques were examined (Baseline, After treatment, and After 5 Days).

**Results:**

The use of the mouthwash containing *GU* extract significantly decreased the clinical values in all parameters of O’Leary index, PI, and GI (*p* < 0.05). Also, after gargling using the mouthwash containing *GU* extract, the antibacterial effect on Gram-positive and Gram-negative bacteria that cause periodontal disease was confirmed to have excellent clinical effects in the inhibition, prevention, and treatment of periodontal disease.

**Conclusion:**

The mouthwash containing *GU* extract can be beneficial in maintaining a healthy periodontal condition and oral hygiene through the reduction of O’Leary index, PI, GI, and periodontal-disease-related bacteria in the oral cavity, which has been proven in clinical applications.

**Supplementary Information:**

The online version contains supplementary material available at 10.1186/s12903-025-06172-2.

## Background

Periodontal disease is an inflammatory disease that causes tooth loss by destroying the alveolar bone and periodontal ligaments as dental plaque accumulates in the gingiva [1]. While periodontal disease is caused by a variety of factors, such as hematologic disease or hormone deficiency, anaerobic bacteria found in dental plaque are known to be the most important cause [2]. Studies on bacteria in dental plaques have been conducted since these findings were first revealed in the late 1960s and early 1970s [3]. So far, it has been discovered that there are approximately 400 species of bacteria in dental plaque, and studies on the distribution of bacteria that cause periodontal diseases are being actively conducted [4]. The two types of periodontal disease are gingivitis and periodontitis. Gingivitis occurs non-specifically because inflammation is confined to the gingiva [5]. This is caused by bacteria such as Gram-positive bacillus *Actinomyces sp. A. naeslundii*, Gram-negative cocci *Veillonella sp. V. parvule*, and others [6]. Since most periodontal disease bacteria are anaerobic bacteria that are sensitive to oxygen, they are distributed in the subgingival plaque, which has a poorer supply of oxygen compared to the supragingival plaque [7]. Therefore, as gingivitis progresses to periodontitis, the Gram-negative bacteria species increase [8], and Gram-negative obligate anaerobes, such as *Porphyromonas sp. P. gingivalis*, *Prevotella sp. P. intermedia*, *Fusobacterium sp. F. nucleatum*, and *Actinobacillus sp. A. actinomycetemcomitans*, are found in the gingivitis sites in which alveolar bone is absorbed and tooth-supporting complex is lost [9]. It has also been reported that, on average, the amount, proportion, and frequency of periodontal disease-causing bacteria, such as *P. gingivalis*,* T. forsythensis* of *Tannerella species*, and *T. denticola* of *Treponema species*, increase in the subgingival area of patients with chronic periodontitis [7]. Many researchers are trying to investigate the correlation between certain periodontal diseases and bacteria.

The progression of periodontal disease can cause not only halitosis, stomatitis, and alveolar bone resorption, but also oral health problems such as tooth loss, which can lead to nutritional imbalance. Furthermore, it is reported that there is a correlation with systemic diseases, such as diabetes and cardiovascular disease, so prevention and early treatment of periodontal disease are crucial [10]. Typical antibiotics for chemotherapeutic treatment used in periodontal treatment include tetracyclines, metronidazole, penicillin, clindamycin, ciprofloxacin, etc [11]. Tetracycline, the most widely used systemic antibiotic, has been demonstrated to be effective for the rapidly progressive periodontitis caused by *A. actinomycetemcomitans* and in inhibiting bone loss and increasing bone regeneration [12]. Its long-term use, however, is limited in terms of results after prolonged use in the expression of antibiotic-resistant bacteria and gastrointestinal disorders [13]. Since these compounds have several problems in preventing oral diseases, studies on oral disease prevention using natural extracts that have few side effects and can be used stably for a long time have recently been increasing [14].

*Glycyrrhiza uralensis* (*GU*, licorice, *Glycyrrhiza glabra*), a perennial plant belonging to the legume family, is harmless to the human body and is widely used as a medicine [15]. The non-toxic effects of *GU* have been reported in many previous studies [16, 17]. It is widely used not only for herbal medicine prescription and edible products in East Asia, mainly in Korea, China, and Japan, but also for licorice candy and sweeteners in western countries, especially in Northern European countries like Finland and Iceland. According to statistics, the Dutch consumes an average of 2 kg of *GU* per person [18]. *GU* has been reported to have excellent effects on diabetes [19], allergy [20], chronic hepatitis [21], and AIDS [22]. It has also been reported that glycyrrhetinic acid, a component of *GU* extract, inhibits plaque deposition by *Streptococcus mutans*, which causes dental caries in oral diseases and inhibits bacterial glycosyltransferase activity [23]. According to a recent study, mouthwash containing *GU* extract demonstrated excellent antibacterial activity through inhibition and effective reduction of bacteria that cause dental caries [24].

However, no studies have been conducted to confirm the clinical antibacterial effect of *GU* extract against bacteria that cause periodontal disease. Consequently, the objective of this study is to the effectiveness of the use of mouthwash containing *GU* extract against the null hypothesis that it would not be effective in reducing periodontal-related indicators and periodontal disease-related bacteria.

## Materials and methods

### Ethical consideration

This study was conducted in accordance with the International Council for Harmonization of Technical Requirements for Pharmaceuticals for Human Use (ICH) guideline. The human study was approved by the Kangwon National University (KNU) Institutional Review Board (KWNUIRB-2020-07-008-001, Chuncheon, South Korea) and WHO International Clinical Trial Registry Platform was registered by clinical trial registration (11/02/2022, registration number: KCT0006998; https://cris.nih.go.kr/cris/search/detailSearch.do/20789). All pertinent information (purpose, procedures, and risks) of this study were explained to all participants. The participants have the freedom to withdraw from the study at any time. A written informed consent form was provided to all participants prior to enrollment in the clinical trial. In addition, we informed all study subjects that there were no potential harms or side effects from using mouthwash, and only those who agreed to this became study participants.

### Study participants

**Fig. 1:**
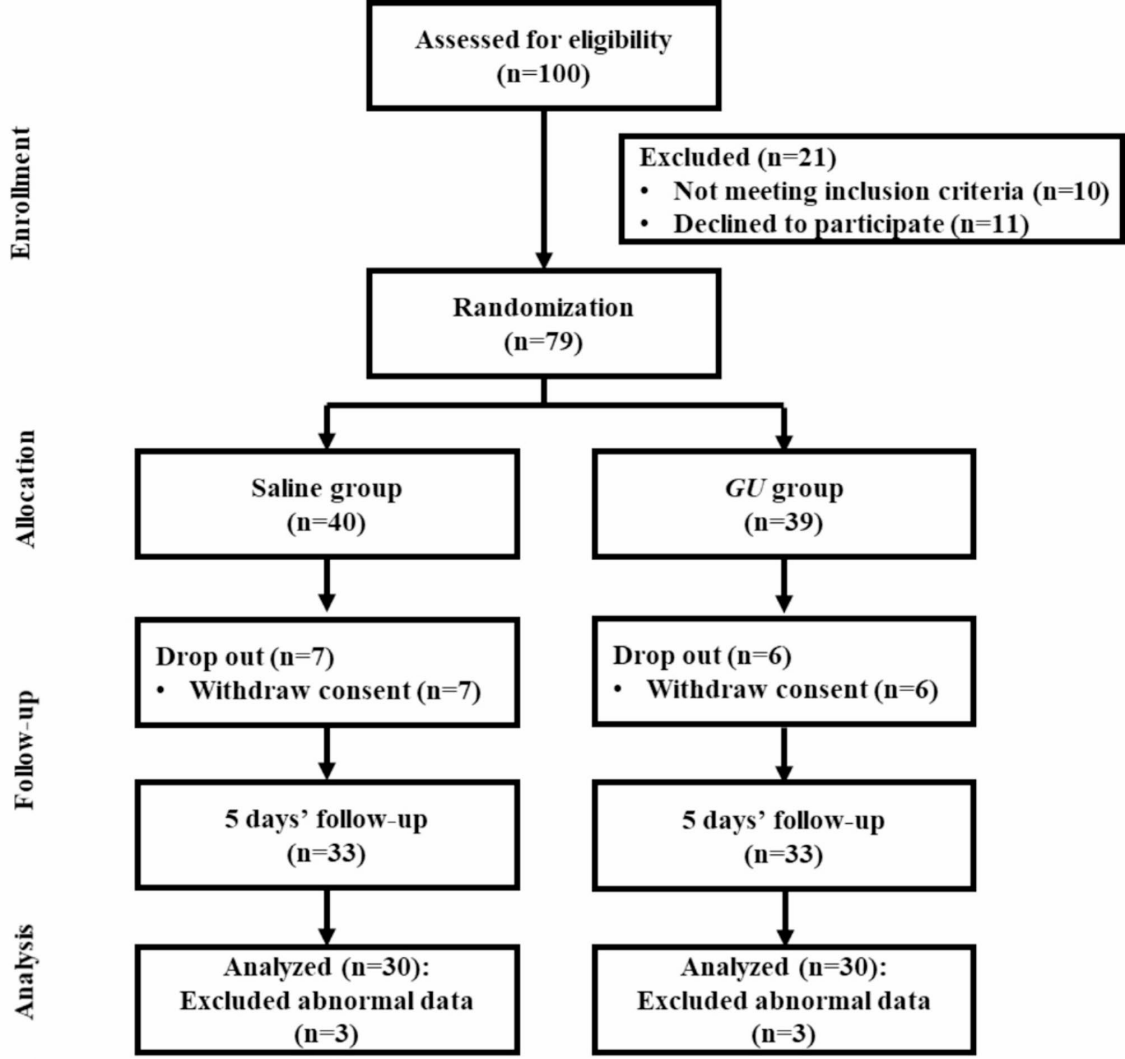
Flow chart of the study

The sample size was calculated using the G*Power 3.1 program. The number of participants needed for the independent t-test with significance level α = 0.05 bilateral test, power = 0.8, and effect size = 0.7 was 68. The initial sample size was planned at 96, which was planned considering a dropout rate of 40%, but a total of 100 participants were actually recruited. The dropout rate was high because the participants were college students or office worker. After excluding 21 subjects who did not meet the inclusion criteria or who refused to participate during the 5 days, a total of 100 subjects were screened and 79 participants were randomly assigned to either the saline gargle group or the *GU* extract gargle group. The final participants for the study consisted of 60 subjects, excluding 13 subjects who did not follow the guidelines for 5 days and 6 subjects who did not complete the final analysis (Fig. 1). The study was conducted similarly to the research method used in Kim and Nam’s study [25].

### Study design and protocol

A dental hygienist with more than 10 years of experience directly explained the objective of the study to the patients who visited M dental clinic in Busan from October 2020 to June 2021. Only those who agreed to participate in the study were included. Among the study subjects, the following were excluded: those with severe dental disease such as periodontitis, dental caries, dry mouth, etc. (patients with periodontal disease who have a stable periodontal condition can participate in the study, and patients with a periodontal pocket depth of 4 mm or more are excluded, patients with enamel caries only are eligible to participate in the study, with the exception of patients with more than one dentine caries); those who are receiving treatment for hepatic disease, renal disease, Sjogren’s syndrome, and rheumatism etc. and with general disease that may cause oral odor those who smoke; those who have been diagnosed with sinusitis and/or rhinitis, those who are taking antibiotics, those with tongue problems such as tongue cancer, glossitis, etc., and those who received scaling within 2 months. The subjects of this study were patients who agreed to complete the questionnaire, who were not included in the exclusion criteria, and had 16 or more remaining teeth. The final 60 patients who met the inclusion criteria were chosen as the study subjects. A randomized, double-blind, controlled clinical trial was conducted. The study was conducted similarly to the research method used in Kim and Nam’s study [25].

### Clinical examination

Two dental hygienists who received oral examination and training from a dentist performed oral scaling to create the same oral conditions. The study began one week after scaling to allow for the recovery period of oral tissue. In order to use the same oral aid products during the study period, the same toothbrush and toothpaste were provided when visiting M dental clinic. The periodontal index was evaluated by selecting three maxillary teeth (#16, #21, #24) and three mandibular teeth (#36, #41, #44). All subjects participating in the study were instructed on toothbrushing education and how to apply mouthwash. The subjects were labeled with light-blocking bottles so that they could not identify which group they belonged to, and the two bottles were randomly distributed. Additionally, before going to bed, children were taught to brush their teeth using the toothbrush and toothpaste provided and then gargle. The study group administered 15 mL of containing 1 mg/mL *GU* (0.015 gm of extract powder/15 ml of distilled water) mouthwash for 30 s before going to sleep, and the control group received 15 mL of saline for 30 s for 5 days [26]. After gargling, drink (water or beverages) or food ingestion was not allowed. This study was conducted to confirm the effect over a relatively short period of 5 days because it excluded subjects with periodontitis with periodontal pocket depth of 4 mm or more. After 5 days, the subjects visited the dentist immediately after waking up and before breakfast without any oral hygiene activities including brushing and gargling, and the indicators were measured. The O’Leary index, plaque index (PI), and gingival index (GI), which are widely used as periodontal status evaluation indices [26–28], were used, and microbiological analysis was performed to identify bacteria related to periodontal disease [30]. The measured data were collected for a total of 3 times from before gargle application (Baseline), immediately after gargle application (Treatment), and 5 days after gargle application (After 5 Days) and was secured by 2 dental hygienists who were trained under the guidance of a dentist. The study was conducted similarly to the research method used in Kim and Nam’s study [25].

### O’Leary index

The O’Leary index measured in this study was performed based on the studies of Lang and Tonetti, which are widely used as oral hygiene examination [27]. The measurement method discolored all teeth in the oral cavity, and then record the degree of adhesion (%) by scoring 1 point if plaque was present on the four tooth surfaces (mesial, efferent, facies, and lingual), and 0 otherwise. The study was conducted similarly to the research method used in Kim and Nam’s study [25].

### PI

In this study, PI of Loe and Silness [28] was used as a standard. The measurement method was to measure plaque accumulation and thickness by applying a discoloration agent to the tooth surface and dividing the tooth surface into two parts. The evaluation criteria were measured from 0 to 3 points. A score of 0 means no calculus, and a score of 1 means that there is a thin layer of plaque attached to the gingival margin that is exposed after lightly scraping it off with a probe or applying tooth staining agent. A score of 2 indicates the presence of moderate calculus visible to the naked eye along the gingival margin, and a score of 3 indicates the presence of thick calculus on the gingival pocket, gingival margin, and tooth surface. The PI score for each tooth was calculated as an average value and proceeded similarly to in Kim and Nam’s study research method [25].

### GI

GI was measured based on the study by Silness and Loe, and was evaluated in four areas of the teeth (proximal, distal, buccal, and lingual sites) [29]. The measurement method was recorded from 0 to 3 points depending on the symptoms of each area. As a measurement standard, normal gingiva was given a score of 0, and mild inflammation with slight color change and swelling, and inflammation without bleeding due to mild irritation was given a point of 1. Moderate inflammation with redness, swelling, and bleeding due to mild irritation was given a score of 2, and severe inflammation with significant redness or swelling, and the possibility of ulceration and spontaneous bleeding, was given a score of 3. The total mean GI for an individual was calculated by adding the values for each tooth. The study was conducted similarly to the research method used in Kim and Nam’s study [25].

### Microbiological analysis

The sample of subgingival microbiota were collected from the gingival sulcus at a total of 4 sites: 2 maxillary teeth (#16 and #21) and 2 mandibular teeth (#36 and #41) from subjects with a pocket depth (PD) of less than 4 mm. #15 paper points were collected for 10 s, placed in a sterilized 1.5 mL microcentrifuge tube. DNA extraction was performed using the AccuPrep Universal RNA Extraction Kit (Bioneer, Daejeon, South Korea). The extraction was performed according to the manufacturer’s instructions. OligoMix (YD Global Life Science Co., Ltd., Seongnam, South Korea) and 3 oligonucleotides (forward primer, reverse primer, and probe) that react specifically to each bacterium were used [29]. In order to prepare the polymerase chain reaction (PCR) sample, 9 µL of OligoMix, 10 µL of 2x probe qPCR mix (Takara Bio Inc., Shiga, Japan), and 1 µL of template DNA were mixed and used on the CFX96 Touch Real- Time PCR Detection System (Bio-Rad, Hercules, USA). PCR initial activation step for 30 s at 95 °C, denaturation for 10 s at 95 °C, and annealing for 30 s at 62 °C with 40 repeated cycles. The cycle threshold (Ct) parameter was calculated from the Bio-Rad CFX Manager Software program (Supplementary Table 1). The study was conducted similarly to the research method used in Kim and Nam’s study [25].

### Statistical analysis

All data were analyzed using SPSS 24.0 for Windows (IBM Corp., Armonk, NY, USA). The demographic characteristics (gender, age, systemic disease) of the subjects participating in this study were analyzed for frequency. An independent t-test was conducted at a 5% significance level in order to verify the significance of the saline gargle group and the *GU* extract gargle group for all measured periodontal-related index and oral bacterial tests. One-way ANOVA was used for the statistical analysis of the Baseline, Treatment, and After 5 Days of gargle application, while Tukey’s test was performed as a post-hoc analysis.

## Results

### Population characteristics

The general characteristics of the study subjects are presented in Table 1. In terms of gender distribution, the control group consisted of 21 women and 9 men, while the test group consisted of 24 women and 6 men, indicating no significant difference between the two groups (*p* > 0.05). The mean age of the subjects was 41.63 ± 10.84 years in the control group and 38.50 ± 10.51 years in the study group, indicating no significant difference between the two groups (*p* > 0.05). No significant differences were also observed between the two groups in terms of systemic disease (*p* > 0.05).

**Table 1:** Characteristics of the subjects in the saline and *GU* groups

Characteristics				*N* (%)		
			Saline		*GU*	*p*-value
^*^Gender	Male		9 (30)		6 (20)	0.761
	Female		21 (70)		24 (80)	
^a^Age (mean ± SD)			41.63 ± 10.84		38.50 ± 10.51	0.573
^*^Systemic disease		No disease	27 (90)		27 (90)	1.000
		Have a disease	3 (10)		3 (10)	

^a^*p*-values are determined by independent t-test. ^*^p-values are determined by chi-square test (*p* < 0.05). Values are means ± standard deviations; significant (in bold)

### Measuring clinical outcomes

Table 2 shows the measurement results of periodontal-disease-related clinical parameters between the saline gargle group and the *GU* extract gargle group. For O’Leary index, PI, and GI, there was no significant difference between the saline gargle group and the *GU* extract gargle group when measured at baseline (*p* > 0.05). However, there were significant differences between the two groups when measured immediately after Treatment and After 5 Days (*p* < 0.05). Upon comparison of the measured periodontal-disease-related parameters for the saline gargle group and the *GU* group, the application of the gargle solution containing the *GU* extract showed a clearly low level, thereby confirming its excellent clinical effect.

**Table 2:** Clinical outcomes observed between the groups

Variables	Group	Mean ± SD			
		Baseline	Treatment	After 5 Days	^*^p-value
O’Leary index	Saline	56.87 ± 11.98^a^	40.62 ± 11.20^b^	41.38 ± 11.39^b^	**0.015**
	*GU*	53.79 ± 13.02^a^	18.07 ± 6.35^b^	21.86 ± 9.41^b^	**0.000**
	^a^*p-*value	0.645	**0.000**	**0.000**	
Plaque index (PI)	Saline	1.88 ± 0.46^a^	1.62 ± 0.52^a^	1.74 ± 0.52^a^	0.624
	*GU*	1.93 ± 0.45^a^	0.68 ± 0.24^b^	0.99 ± 0.42^b^	**0.000**
	^a^*p-*value	0.832	**0.000**	**0.000**	
Gingival index (GI)	Saline	1.49 ± 0.50^a^	1.20 ± 0.52^a^	1.35 ± 0.39^a^	0.460
	*GU*	1.50 ± 0.51^a^	0.40 ± 0.15^b^	0.66 ± 0.25^b^	**0.000**
	^a^*p-*value	0.980	**0.000**	**0.000**	

^a^*p*-values are determined by independent t- test. * *p*-values are determined by one-way ANOVA and Tukey’s test (*p* < 0.05). Values are means ± standard deviations; significant (in bold)

For the application time from Baseline, Treatment, and After 5 Days, the saline gargle group showed a significant difference in time series only in the O’Leary index (*p* < 0.05). On the other hand, it was confirmed that the *GU* group showed significantly lower values in all clinical indicators of O’Leary index, PI, and GI as the application time increased (*p* < 0.05).

### Microbiological analysis of gram-positive bacteria in subgingival plaques

Table 3 shows the Gram-positive bacterial data in subgingival plaques. Three types of bacteria (*Parvimonas micra [P. micra]*, *Staphylococcus aureus [S. aureus]*, and *Eubacterium nodatum [E. nodatum]*) were observed in both groups. In terms of the comparison of the saline gargle group and the *GU* extract gargle group for *P. micra*, there was a significant difference between the two groups at After 5 Days in the maxilla (*p* < 0.05) as well as at Baseline and Treatment, but did not show a significant difference between the two groups After 5 Days (*p* > 0.05). For *S. aureus*, there was a significant difference at Treatment in the maxilla, and at After 5 Days in the mandible (*p* < 0.05). *E. nodatum* showed a significant difference between the two groups at Treatment in both maxilla and mandible (*p* < 0.05).

**Table 3:** Gram-Positive Bacteria measurements in subgingival plaque

Variables		Group	Mean ± SD			
			Baseline	Treatment	After 5 Days	^*^p-value
*Parvimonas micra*	Maxilla	Saline	2023.30 ± 321.36^a^	2292.90 ± 1954.72^a^	2666.23 ± 1564.12^a^	0.762
		*GU*	8819.40 ± 12748.32^a^	2392.33 ± 2873.29^a^	164.56 ± 192.65^a^	0.098
		^a^*p-*value	0.155	0.939	**0.000**	
	Mandibular	Saline	2117.63 ± 947.87^a^	2623.70 ± 2628.21^a^	2704.43 ± 2532.02^a^	0.852
		*GU*	476.70 ± 440.00^a^	119.89 ± 140.94^a^	1288.56 ± 1788.70^a^	0.117
		^a^*p-*value	**0.000**	**0.014**	0.225	
*Staphylococcus aureus*	Maxilla	Saline	24.57 ± 25.41^a^	0.00 ± 0.00^b^	8.47 ± 13.52^a,b^	**0.018**
		*GU*	12.10 ± 18.46^a^	1.58 ± 1.82^a^	3.00 ± 4.58^a^	0.163
		^a^*p-*value	0.282	**0.037**	0.301	
	Mandibular	Saline	6.80 ± 8.70^a^	2.17 ± 3.16^a^	34.00 ± 33.10^b^	**0.005**
		*GU*	15.30 ± 20.63^a^	0.00 ± 0.00^b^	0.00 ± 0.00^b^	**0.024**
		^a^*p-*value	0.305	0.070	**0.009**	
*Eubacterium nodatum*	Maxilla	Saline	464.23 ± 502.27^a^	467.60 ± 698.19^a^	466.43 ± 440.33^a^	1.000
		*GU*	140.90 ± 199.54^a^	13.56 ± 19.54^a^	1.33 ± 1.92^a^	0.055
		^a^*p-*value	0.110	0.086	**0.007**	
	Mandibular	Saline	435.50 ± 492.80^a^	155.00 ± 229.08^a^	154.83 ± 181.07^a^	0.171
		*GU*	435.50 ± 531.03^a^	6.33 ± 5.34^b^	0.67 ± 0.96^b^	**0.015**
		^a^*p-*value	1.000	0.086	**0.027**	

^a^*p*-values are determined by independent t- test. * *p*-values are determined by one-way ANOVA and Tukey’s test (*p* < 0.05). Values are means ± standard deviations; significant (in bold)

In addition, for the change in the application time from the baseline, there was no significant difference in both the saline gargle group and the *GU* extract gargle group for *P. micra*. In the saline gargle group, there was a significant difference between the maxilla and the mandible for *S. aureus* (*p* < 0.05). There was also a significant difference compared to the baseline at Treatment in the maxilla and at After 5 Days in the mandible. In the *GU* extract gargle group, there was a significant difference only in the mandible for both strains of *S. aureus* and *E. nodatum* (*p* < 0.05), thereby showing a clear difference from Treatment to After 5 Days compared to the Baseline.

### Microbiological analysis of gram-negative bacteria in subgingival plaques

Table 4 shows the Gram-negative bacterial data in subgingival plaques. Eight types of bacteria (*P. gingivalis*, *T. forsythia*, *T. denticola*, *F. nucleatum*, *P. intermedia*, *P. nigrescens*, *E. corrodens*, and *C. rectus*) were observed in both groups. In *P. gingivalis* and *T. forsythia*, there was a significant difference between the saline gargle group and the *GU* extract gargle group at After 5 Days for both maxilla and mandible (*p* < 0.05). For *T. denticola*, there was a significant difference between the two groups only in the mandible at After 5 Days (*p* < 0.05). For *F. nucleatum*, there was a significant difference at After 5 Days in the maxilla and immediately after Treatment in the mandible (*p* < 0.05). For *P. intermedia*, there was a significant difference between the two groups in both maxilla and mandible at After 5 Days (*p* < 0.05). For *P. nigrescens*, there was a significant difference at After 5 Days and Treatment in the maxilla (*p* < 0.05), and at After 5 Days in the mandible (*p* < 0.05). *E. corrodens* showed a significant difference at After 5 Days in the mandible, while *C. rectus* showed a significant difference at After 5 Days in the maxilla (*p* < 0.05).

**Table 4:** Gram-Negative Bacteria measurements in subgingival plaque

Variables		Group	Mean ± SD			
			Baseline	Treatment	After 5 Days	^*^p-value
*Porphyromonas gingivalis*	Maxilla	Saline	44.83 ± 41.56^a^	64.60 ± 93.58^a^	67.73 ± 82.54^a^	0.820
		*GU*	12.80 ± 17.70^a^	2.48 ± 1.87^a^	0.00 ± 0.00^a^	0.059
		^a^*p-*value	0.059	0.080	**0.032**	
	Mandibular	Saline	15.57 ± 16.86^a^	16.00 ± 23.91^a^	22.37 ± 23.51^a^	0.794
		*GU*	2318.50 ± 3191.03^a^	9.19 ± 6.89^b^	3.11 ± 4.48^b^	**0.034**
		^a^*p-*value	0.058	0.460	**0.035**	
*Tannerella forsythia*	Maxilla	Saline	1780.80 ± 2391.14^a^	26542.40 ± 40494.22^a^	27723.00 ± 37016.48^a^	0.208
		*GU*	741.20 ± 1130.81^a^	0.00 ± 0.00^a^	0.00 ± 0.00^a^	0.061
		^a^*p-*value	0.288	0.083	**0.049**	
	Mandibular	Saline	3611.30 ± 4557.39^a^	3531.30 ± 5387.50^a^	4037.07 ± 4096.99^a^	0.975
		*GU*	3611.30 ± 4557.39^a^	69.89 ± 100.72^b^	0.00 ± 0.00^b^	**0.019**
		^a^*p-*value	1.000	0.089	**0.011**	
*Treponema denticola*	Maxilla	Saline	2424.38 ± 3117.10^a^	23871.20 ± 36379.62^a^	24004.53 ± 35424.37^a^	0.279
		*GU*	689.10 ± 965.61^a^	45.33 ± 65.33^b^	0.00 ± 0.00^b^	**0.046**
		^a^*p-*value	0.161	0.083	0.074	
	Mandibular	Saline	1726.50 ± 1475.20^a^	2489.10 ± 3783.94^a^	3132.20 ± 3464.00^a^	0.678
		*GU*	726.50 ± 741.78^a^	557.56 ± 774.77^a^	0.00 ± 0.00^a^	0.082
		^a^*p-*value	0.105	0.181	**0.019**	
*Fusobacterium nucleatum*	Maxilla	Saline	444876.84 ± 461508.19^a^	466566.20 ± 490296.39^a^	45986.03 ± 393786.86^a^	0.996
		*GU*	326600.70 ± 434523.27^a^	123548.04 ± 107921.51^a^	81202.22 ± 100773.54^a^	0.196
		^a^*p-*value	0.627	0.071	**0.018**	
	Mandibular	Saline	209718.43 ± 63748.55^a^	282978.20 ± 269859.41^a^	287408.20 ± 141062.37^a^	0.644
		*GU*	144385.10 ± 93333.44^a^	74000.11 ± 66920.29^a^	222281.11 ± 308532.02^a^	0.352
		^a^*p-*value	0.119	**0.048**	0.604	
*Prevotella intermedia*	Maxilla	Saline	2413.59 ± 4181.41^a^	3698.00 ± 5572.97^a^	3258.00 ± 2832.85^a^	0.847
		*GU*	373.70 ± 550.59^a^	759.78 ± 1043.27^a^	1.44 ± 2.08^a^	0.127
		^a^*p-*value	0.203	0.167	**0.004**	
	Mandibular	Saline	296.69 ± 248.70^a^	269.80 ± 282.97^a^	244.17 ± 223.44^a^	0.924
		*GU*	220.60 ± 226.76^a^	142.00 ± 201.24^a^	1.56 ± 2.24^a^	0.069
		^a^*p-*value	0.541	0.335	**0.006**	
*Prevotella nigrescens*	Maxilla	Saline	17307.90 ± 24679.76^a^	17465.80 ± 17501.00^a^	18269.13 ± 17318.51^a^	0.995
		*GU*	16,731 ± 24301.87^a^	257.50 ± 120.13^a^	365.11 ± 462.47^a^	0.061
		^a^*p-*value	0.964	**0.012**	**0.008**	
	Mandibular	Saline	1758.81 ± 1785.78^a^	1635.20 ± 1466.58^a^	1580.53 ± 1235.51^a^	0.975
		*GU*	2389.40 ± 2471.53^a^	1688.78 ± 1876.61^a^	68.13 ± 74.77^a^	0.075
		^a^*p-*value	0.592	0.952	**0.002**	
*Eikenella corrodens*	Maxilla	Saline	205.00 ± 215.82^a^	189.30 ± 277.63^a^	234.83 ± 266.14^a^	0.940
		*GU*	105.00121.53^a^	11.89 ± 12.26^a^	304.40 ± 464.41^a^	0.139
		^a^*p-*value	0.275	0.091	0.723	
	Mandibular	Saline	114.73 ± 139.40^a^	147.00 ± 130.41^a^	108.67 ± 106.06^a^	0.817
		*GU*	81.40 ± 94.79^a^	62.89 ± 90.63^a^	0.00 ± 0.00^a^	0.097
		^a^*p-*value	0.590	0.160	**0.008**	
*Campylobacter rectus*	Maxilla	Saline	6779.60 ± 10366.56^a^	6786.50 ± 10353.78^a^	7812.70 ± 9297.14^a^	0.974
		*GU*	179.60 ± 274.01^a^	0.00 ± 0.00^a^	0.00 ± 0.00^a^	0.061
		^a^*p-*value	0.092	0.083	**0.029**	
	Mandibular	Saline	194.20 ± 308.83^a^	0.00 ± 0.00^a^	89.73 ± 125.51^a^	0.154
		*GU*	124.70 ± 190.25^a^	0.00 ± 0.00^a^	0.00 ± 0.00^a^	0.052
		^a^*p-*value	0.602	-	0.060	

^a^*p*-values are determined by independent t- test. * *p*-values are determined by one-way ANOVA and Tukey’s test (*p* < 0.05). Values are means ± standard deviations; significant (in bold)

For the change by time series from the baseline to the application of the gargle solution for 5 days, the saline gargle group showed no significant difference for all 8 types of bacteria detected in the baseline (*p* > 0.05). On the other hand, the *GU* extract gargle group showed a marked difference for *P. gingivalis* and *T. forsythia* in the mandible and for *T. denticola* in the maxilla until After 5 Days from the Baseline (*p* < 0.05).

## Discussion

Since the best way to prevent the occurrence and progression of periodontal disease is to remove the dental plaque, chemical methods (e.g., antibacterial agents, antibiotics, fluoride agents, and enzymes) are being used. However, various side effects have been reported with this method, and there has recently been a growing interest and research on the effectiveness of removing plaque using natural agents and the treatment of early periodontal disease [31]. Moon et al. reported that *Brachypodium sylvaticum* (BS) extract has excellent antibacterial and residual antibacterial effects against strains that cause oral diseases [32]. Various authors also reported that the following substances are effective against the bacteria that cause periodontal diseases: Lee et al. reported the essential oil of *Artemisia lavandulaefolia* [33], Yoon et al. reported unripe apple [34], and Jang et al. reported *Coptis chinensis* [35]. *GU* has already demonstrated antibacterial effects on antibiotic-resistant strains [36], and its inhibiting effects on bacteria that cause dental caries in oral diseases [23]. *GU* has also been shown to be effective in the treatment of periodontal disease [37]. Attempts are being made to clinically utilize the natural materials with antibacterial effects through ongoing research. This study confirmed the clinical applicability and possibility of *GU* extract to be used as an antibacterial material for the prevention of periodontal disease progression through a randomized, double-blind, -controlled clinical trial.

This study investigated the changes in O’Leary index, PI, and GI, which are representative parameters of periodontitis. Compared to saline gargle, the gargle containing *GU* extract showed a clear decrease from immediately after gargle application (Treatment) to 5 days after gargle application (After 5 Days). In addition, the change in oral condition after using the mouthwash containing *GU* extract for 5 days showed that the O’Leary index, PI, and GI index were low. This effect on clinical parameters was faster than in a previous study that reported the clinical parameters of GI and PI decreased after using a mouthwash containing green tea extract for 2 weeks [38]. Listerine gargle has been reported to reduce PI and GI by approximately 50% in a 6-week study [39]. The effects of the gargle solution with *GU* extract showed a decrease in O’Leary index by 40.43%, PI by 51.29%, and GI by 44%, thereby confirming that its effect was similar to the result of the study using Listerine. A study using a 20% concentration (2 g powder/10 mL distilled water) liquorice root extract mouth rinse [40] reported that the average PI and GI scores statistically decreased after 15 days of observation, but the chlorhexidine group showed a greater decrease. This suggests that liquorice root extract limits plaque accumulation and gingival inflammation. Therefore, this demonstrates that *GU* extract is effective impromoting oral hygiene.

Furthermore, this study investigated the changes in the Gram-positive and Gram-negative bacteria, which cause periodontal disease, after use of the mouthwash containing *GU* extract. After using the mouthwash for 5 days, marked changes in the Gram-positive anaerobic bacteria were observed in *P. micra* (maxilla), *S. aureus* (mandibular), and *E. nodatum* (maxilla and mandible) compared to the saline gargle group. Among the significant changes, *S. aureus* and *E. nodatum* were effectively reduced after using the mouthwash containing *GU* extract. *S. aureus* is known as a bacterium that requires attention because it is commonly found in the sacs of peri-implantitis, thus creating an oral environment in which high-risk bacteria for periodontal disease can proliferate and systemically cause endocarditis and osteomyelitis [41]. Based on the results of this study, *S. aureus* showed significant changes in the maxilla and mandible when saline was applied. However, bacteria were not detected in the subgingival plaques in the mandible from Treatment to After 5 Days when the mouthwash containing *GU* was used. *E. nodatum*, which is closely related to periodontitis by inducing alveolar bone reduction and chronic periodontitis [42], confirmed that the bacteria effectively decreased in the subgingival plaques in the mandible from immediately after gargle application to 5 days after gargle application when the mouthwash containing *GU* was used. Based on the above results, the use of the mouthwash containing *GU* extract against Gram-positive bacteria related to periodontal disease is thought to lead to periodontal disease prevention.

Upon continuous use of the mouthwash containing *GU* extract for 5 days, among Gram-negative anaerobic bacteria, *P. gingivalis* (maxilla and mandible), *T. forsythia* (maxilla and mandible), *T. denticola* (mandible), *F. nucleatum* (maxilla), *P. intermedia* (maxilla and mandible), *P. nigrescens* (maxilla and mandible), *E. corrodens* (mandible), and *C. rectus* (maxilla) showed significant differences. This confirmed that the use of the mouthwash containing *GU* extract is an excellent natural material for the improvement of periodontal disease.

Since *P. gingivalis*,* T. denticola*, and *T. forsythia* are abundant in patients with periodontal disease, these bacteria, also known as “red complex”, are recognized as key causative factors of periodontal disease [43]. Their presence is commonly used to check the periodontal condition in studies related to periodontal diseases. Among these, lipopolysaccharide (LPS) of *P. gingivali*, a representative bacterial species that causes periodontal disease, has been found to have an effect on periodontal tissue regeneration by inhibiting the development of osteoblasts and the mineralization of periodontal ligament stem cells. It also causes destruction of periodontal tissue by helping bacterial colonization in the periodontal pocket through the proteolytic enzyme [44]. In this study, following the use of the mouthwash containing *GU* extract, *P. gingivalis* was no longer found in the maxilla, although the change was not statistically significant. Furthermore, the level of *P. gingivali*s decreased immediately after gargle application and maintained for 5 days after gargle application. *T. forsythia* and *T. denticola* are considered important in the diagnosis and treatment of periodontal disease because they are simultaneously detected in areas affected by periodontal disease [45]. It has been reported that *T. forsythia* is associated with early periodontitis or peri-implantitis, and it is systematically associated with diabetes, hypertension, myocardial infarction, arteriosclerosis, coronary artery stenosis, obesity, and rheumatoid arthritis [46]. *T. denticola* causes periodontal disease by adhering to gingival epithelial cells and inducing cytokine production in gingival fibroblasts. This has been linked to early periodontitis, ulcerative gingivitis, and acute periodontitis, and systematically related to coronary artery thrombosis, syphilis (bacterial vaginitis), and others [47]. As a result of using the mouthwash containing *GU* extract for 5 days, subgingival plaque bacteria were not found in the mandible for *T. forsythia* and in the maxilla for *T. denticola*. The results indicate that *GU* extract can be valuably used not only for the prevention and suppression of periodontal disease in the oral cavity, but also for the treatment of periodontal disease.

*E. corrodens*, which destroys periodontal tissue, causes bone resorption through polymicrobial infection and systematically causes arteriosclerosis, endocarditis, meningitis, respiratory infection, osteomyelitis, etc [4]. *C. rectus*, which is known to cause periodontal pockets and periodontal disease as well as systematically cause fever, headache, muscle pain, diabetes, spontaneous abortion (placenta damage and inhibition of fetal growth), food poisoning, etc [4] did not show a significant difference from the baseline. *C. rectus*, however, was not found in the subgingival plaques after using the mouthwash containing *GU* for 5 days. Despite this bacterial reduction effect, our study has several limitations. First, although this study was a relatively rigorous randomized, double-blind, controlled clinical trial study, the Hawthorne effect could not be ruled out. Second, because our study lasted 5 days, there is a possibility of recolonization in the periodontal pockets. Therefore, more long-term clinical evaluation is needed, and research should be conducted to confirm the safety effect to help the public’s oral health.

Despite these limitations, our study confirmed that *GU* extract has an excellent antibacterial effect against bacteria that cause periodontal disease in terms of its inhibitory effect on Gram-positive and Gram-negative bacteria. These results are very significant in that they are the first results that have not been reported so far. In addition, its practicality as a new material derived from nature was confirmed through clinical trial on people rather than vitro study.

Based on the above results, the use of *GU* extract is expected to suggest a new strategy for preventing and treating periodontal disease, since it exhibits antibacterial activity by suppressing the expression of periodontal disease–causing bacteria. Therefore, the mouthwash containing *GU* extract will serve as a natural mouthwash with excellent antibacterial effect that can prevent periodontal disease and contribute to oral health promotion.

## Conclusion

The use of mouthwash containing *GU* extract can be recommended as an auxiliary oral hygiene product to maintain healthy periodontal condition. In particular, *GU* extract is a biocompatible alternative material and is expected to be widely used as a mouthwash to prevent and improve the oral health of the public.

## Electronic supplementary material

Below is the link to the electronic supplementary material.

Supplementary Material 1

## Data Availability

The data sets generated and/or analyzed during the current study are not publicly available for reasons of personal and organizational integrity but are available from the corresponding author on reasonable request.

## Abbreviations

*GU*: *Glycyrrhiza uralensis*
Baseline: Before gargle application
Treatment: Immediately after gargle application
After 5 Days: 5 days after gargle application
PI: Plaque index
GI: Gingival index
*P. micra*: *Parvimonas micra*
*S. aureus*: *Staphylococcus aureus*
*E. nodatum*: *Eubacterium nodatum*
*P. gingivalis*: *Parvimonas micra*
*T. forsythia*: *Tannerella forsythia*
*T. denticola*: *Treponema denticola*
*F. nucleatum*: *Fusobacterium nucleatum*
*P. intermedia*: *Prevotella intermedia*
*P. nigrescens*: *Prevotella nigrescens*
*E. corrodens*: *Eikenella corrodens*
*C. rectus*: *Campylobacter rectus*
