# Supplementary material for: Effectiveness of Glycyrrhiza uralensis extract on periodontal pathogens: a randomized controlled clinical trial
Source: BMC Oral Health. 2025 May 24;25:783. doi: 10.1186/s12903-025-06172-2 (PMC12103038; doi:10.1186/s12903-025-06172-2)
Supplement: Supplementary file 1 — Supplementary Material 1 [file 12903_2025_6172_MOESM1_ESM.docx]

**Supplementary Materials**

The supplementary materials contain one tables. Supplementary Table 1: Primers and Probes Used in the Real-Time PCR Assays.

| **Supplementary Table 1.** Primers and Probes Used in the Real-Time PCR Assays | | | |
| --- | --- | --- | --- |
| **Bacteria** | **Target genes** | **Primers/Probe sets** | Amplicon size (bp) |
| *Parvimonas micra* | 16S ribosomal RNA gene | 5′-GAGGAATACCGGTGGCGAAG-3’  5′-GGCACCGAGATTTGACTCCC-3’  5′-FAM-GGTACGAAAGCGTGGGGAGCA-BHQ1–3’ | 148 |
| *Staphylococcus aureus* | Clumping factor A (clfA) gene | 5′-GCGCAAGTAACGAAAGCAAAA-3’  5′-GATTTTGCGCCACACTCGTT-3’  5′-FAM-TGCTGCACCTAAAACAGACGACACA-BHQ1–3’ | 132 |
| *Eubacterium nodatum* | Hypothetical protein | 5′-TGCTTGCCGGTGACTTAGGA-3’  5′-AAACCGGGCTCAACAACCAT-3’  5′-Texas Red-TTGAGGAGCCGGTGACTTTGG-BHQ2–3’ | 130 |
| *Porphyromonas gingivalis* | Hemagglutinin (phg) gene | 5′-ACACGGTGTATCGTGACGGC-3’  5′-GCCGGCTGCGTACTTAACCT-3’  5′-HEX-CGACCTACCGCGATGCAGGA-BHQ1–3’ | 119 |
| *Tannerella forsythia* | Karilysin protease gene | 5′-TGGCAAATCGCTCATCATCC-3’  5′-TTCCATGTTCCCCAACCACA-3’  5′-Texas Red-CCATTAAGCCCATTGCCCGG-BHQ2–3’ | 140 |
| *Treponema denticola* | Oligopeptidase B (opdB) gene | 5′-AGAAAGGCTTTGGGCGACAG-3’  5′-GCTGGAGCCGTAGCTTCCAT-3’  5′-Cy5-CGGGTCCTCACCCGCTCTTC-BHQ2–3’ | 127 |
| *Fusobacterium nucleatum* | 16S ribosomal RNA gene | 5′-GGCTGTCGTCAGCTCGTGTC-3’  5′-CTCATCGCAGGCAGTATCGC-3’  5′-FAM-AACGAGCGCAACCCCTTTCG-BHQ1–3’ | 114 |
| *Prevotella intermedia* | Hemagglutinin (phg) gene | 5′-CACACGCTGGCGAAACCTAC-3’  5′-CACGTGGCGTTGCTTCTTTC-3’  5′-HEX-CCGAAGATGCGCCGTTGAAC-BHQ1–3’ | 143 |
| *Prevotella nigrescens* | Gyrase subunit B (gyrB) gene | 5′-AGCAAGCTGTAGGCGAGGCT-3’  5′-GCTGAACACTTTCGCGTGCT-3’  5′-Texas Red-GCTCGTATTGCAGCCCGCAA-BHQ2–3’ | 132 |
| *Eikenella corrodens* | Proline iminopeptidase (pip) gene | 5′-GCCAACTGCTGCTGGAAGTG-3’  5′-GCCGCTGATTTCGGAGAGTT-3’  5′-HEX- ACAGCCATCGGCACAGGCAT-BHQ1–3’ | 110 |
| *Campylobacter rectus* | GroEL gene | 5′-AAATTTAAGCGGCGACGAGG-3’  5′-TCCTTGCTCACGCTTACGGA-3’  5′-HEX-GGCTTTGACGCGGGCGTAGT-BHQ1–3’ | 132 |
